# Editorial: Deciphering macrophage polarization/transition in human inflammatory disease and cancer

**DOI:** 10.3389/fimmu.2026.1804398

**Published:** 2026-03-04

**Authors:** Peter Mu-Hsin Chang, Nai-Jung Chiang, Stefania Fiorcari, Yu-Chan Chang

**Affiliations:** 1 Department of Oncology, Taipei Veterans General Hospital, Taipei, Taiwan; 2 Department of Medical and Surgical Sciences, Section of Hematology, University of Modenaand Reggio Emilia, Modena, Italy; 3 Department of Biomedical Imaging and Radiological Imaging, National Yang Ming Chiao Tung University, Taipei, Taiwan

**Keywords:** applications, cancer, inflammation, macrophage, polarization

Macrophages are highly adaptable innate immune cells that respond dynamically to environmental cues by adopting various functional states which play a crucial role in maintaining tissue homeostasis, regulating inflammation, and influencing cancer progression. Rather than existing as distinct M1 or M2 populations, macrophages occupy a spectrum of activation states, which are influenced by transcriptional, metabolic and signaling networks. This concept has been emphasized in recent integrative reviews of kidney disease and cancer biology (Ma et al.; Zhou et al.). Dysregulated macrophage polarization is a key driver of chronic inflammatory diseases and tumor progression; however, the mechanisms governing macrophage transition and their translational relevance are not fully understood. This Research Topic brings together 11 articles that collectively advance our understanding of macrophage polarization and transition in human inflammatory diseases and cancer.

Several studies emphasize the clinical and prognostic relevance of macrophage heterogeneity in cancer. Cao et al. used a combination of transcriptomic profiling and computational modelling to identify gene signatures associated with macrophages that can be used to predict patient outcomes in clear cell renal cell carcinoma. This highlights the prognostic and functional importance of tumor-associated macrophages (TAMs). Further complementary analyses demonstrate that macrophage polarization influences tumor progression, immune suppression and metastatic behavior in malignancies such as oral squamous cell carcinoma Dong et al., thereby reinforcing the role of TAMs as key regulators of the tumor microenvironment and as potential therapeutic targets.

Beyond oncology, this Research Topic highlights the important role of macrophages in various inflammatory and tissue-specific contexts. For example, Zhao et al. demonstrate that the long non-coding RNA NRIR promotes M1 macrophage polarization via the RSAD2/NF-κB axis in peri-implantitis, thereby linking macrophage-driven inflammation to impaired osteogenesis. Shen et al. identify the adaptor protein Grb2 as a key mediator of macrophage activation in acute pancreatitis, and Wang et al. use single-cell transcriptomics to reveal distinct macrophage-associated inflammatory niches in granulomatous lobular mastitis. More broadly, Bamahel et al. present a bibliometric and systematic analysis of research into macrophage polarization in osteoarthritis, identifying emerging trends and emphasizing significant knowledge gaps.

Importantly, this Research Topic also explores the therapeutic modulation of macrophage polarization. Scuoppo et al. demonstrate that the pharmacological targeting of C/EBPβ with the antagonist peptide lucicebtide (ST101) can reprogram macrophages towards pro-inflammatory and anti-tumor phenotypes, thereby enhancing immune responses in cancer models. In a related brief report, Brumfield et al. identify amyloid precursor-like protein 2 as a regulator of macrophage differentiation and M1/M2 balance. Furthermore, Ruan et al. provide mechanistic evidence linking macrophage polarization dynamics to inflammatory disease progression, thereby reinforcing the concept that macrophage states are dynamically regulated and susceptible to therapeutic intervention.

Collectively, the contributions to this Research Topic reinforce several unifying principles. Macrophage polarization is dynamic and context-dependent, with close links to local microenvironmental cues. Advanced methodologies, including single-cell transcriptomics, integrative bioinformatics and functional validation, are essential for understanding macrophage heterogeneity and translating mechanistic insights into clinical applications. Integrating basic, translational, and clinical perspectives, this Research Topic provides a comprehensive overview of the latest advances and challenges in macrophage polarization research, setting the scene for future macrophage-targeted therapeutic strategies.

